# The spin structures of interlayer coupled magnetic films with opposite chirality

**DOI:** 10.1038/s41598-018-20800-8

**Published:** 2018-02-05

**Authors:** S. P. Kang, N. J. Kim, H. Y. Kwon, J. W. Choi, B. C. Min, C. Won

**Affiliations:** 1 Department of Physics, Kyung Hee University, Seoul, 02447 Korea; 2 Center for Spintronics Research, Korea Institute of Science and Technology, Seoul, 02792 Korea

## Abstract

Using Monte-Carlo simulations and micromagnetic simulations, we reveal how the spin structural correlation and the skyrmion dynamics are affected by the interlayer coupling in a chiral magnetic bilayer system, in which the two layers have opposite chirality. The interaction through interlayer coupling between chiral magnetic structures influences the static and dynamics properties profoundly. The competition between the Dzyaloshinskii-Moriya interaction and the interlayer interaction allows multiple magnetic structures to be energetically stable, which includes sole skyrmion states (skyrmion appears in only one of the layers) and skyrmion pair states (coupled skyrmions in top and bottom layers). When current driven spin transfer torques are applied to each state, the sole skyrmion state is mainly propelled by a spin transfer torque causing the skyrmion hall effect, but the skyrmion pair state is propelled by a torque from skyrmion-skyrmion interaction and not influenced by the skyrmion hall effect. Also upon application of an external magnetic field, we found the skyrmions in a skyrmion pair state extinguish in an exclusive way, as the annihilation of a skyrmion in one of the layers stabilizes the once paired skyrmion in the other layer, i.e. the skyrmion lattice sites have only one skyrmion in either layer.

## Introduction

Spintronics using magnetic skyrmions has been intensively studied in recent years^1–9^. Magnetic skyrmions are considered emerging candidates of a magnetic information unit due to their topological stability and good mobility. A skyrmion is topologically stable because spatially twisted magnetization provides a topological number and it cannot be destroyed or generated without a topological transition. It is also highly mobile with small spin currents since its current induced translational motion does not accompany the dissipation of energy^10,11^. Extensive researches have been focused on manipulating the properties and dynamics of skyrmions^12–14^.

Magnetic chiral structures, including skyrmions, are generated by Dzyaloshinskii-Moriya(DM) interaction which twists directions of spins in an interacting spin pair^15,16^. The scale of a chiral structure is determined by the strength of DM interaction and the chirality of the structure is determined by the direction of DM vector that is related to the symmetry of the system^17,18^. Therefore, the strength and direction of chirality can be controlled by making an appropriate structure or interface^19–21^. One of the methods to control the chirality is using interlayer coupled magnetic bilayer systems. In such systems, the coupling between the magnetic layers shows oscillatory behavior in which there can be ferromagnetic and antiferromagnetic coupling depending on the interlayer thickness. The interlayer coupling can provide an effective field which can replace an external field required to produce skyrmion structure^22–24^. Also, for skyrmion pairs in antiferromagnetically coupled systems, the skyrmion Hall effect(SkHE) can be eliminated due to the cancelling skyrmion numbers of the paired skyrmions, while preserving the skyrmion properties^25,26^.

In this study, we discuss how interlayer coupling affects the spin structure of a bilayer system in which the two layers have opposite chirality (opposite DM interaction). The opposite chirality twists the chiral structures in each layer in opposite directions, so that the DM interaction prefers to break the spin correlation between two layers. However, the interlayer coupling acts to build spin correlation against the chirality. Therefore, the competition between the DM interaction energy and the interlayer coupling energy results in complex spin structures, in which sole skyrmion states and paired skyrmion states coexist. The sole-skyrmion state is a skyrmion appearing in only one of the layers, and the paired-skyrmion state is the coupled skyrmions of opposite skyrmion number in top and bottom layer. These skyrmion states are energetically stable without an external field, because the interlayer coupling provides an effective field. The dynamics of the sole skyrmion and the skyrmion pair are distinctive; When the STT in two layers are identical, the sole skyrmion is mostly influenced by the adiabatic STT and exhibit the SkHE, while the skyrmion pair is propelled by the non-adiabatic spin transfer torque(STT) without SkHE.

With an external magnetic field, skyrmion lattice composed of skyrmion pairs forms. It is composed of two skyrmions with same skyrmion number. The skyrmions in the paired skyrmion states are annihilated in a special exclusionary way, showing potential for use of these systems in spin logic circuit.

## Model

We studied the bilayer system of which each layer has DM interaction of opposite chirality. The total energy of the system studied is described by the following equation;

1 $$\begin{array}{c}U=-{J}_{1}\sum _{ < i,j > }{{\bf{S}}}_{1,i}\cdot {{\bf{S}}}_{1,j}-{J}_{2}\sum _{ < i,j > }{{\bf{S}}}_{2,i}\cdot {{\bf{S}}}_{2,j}-{J}_{{\rm{int}}}\sum _{i}{{\bf{S}}}_{1,i}\cdot {{\bf{S}}}_{2,i}\\ \quad \quad -{{\bf{D}}}_{1,ij}\cdot \sum _{ < i,j > }{{\bf{S}}}_{1,i}\times {{\bf{S}}}_{1,j}-{{\bf{D}}}_{2,ij}\cdot \sum _{ < i,j > }{{\bf{S}}}_{2,i}\times {{\bf{S}}}_{2,j}-\sum _{i}h\cdot ({S}_{1,i,z}+{S}_{2,i,z})\end{array}$$

where, **S** is a dimensionless and normalized vector, *J* the exchange constant, **D**_*ij*_ is the DM vector, *h* is the external field perpendicular to the film and 1,2 indexes each layer. In our study, we set **D**_1, *ij*_ = −**D**_2, *ij*_ to implement opposite chirality and set *J*_int_ to be negative for the case of antiferromagnetic coupling between two layers. The cases for ferromagnetic coupling (*J*_int_ > 0) can be discussed by substituting **S**_2_→−**S**_2_ in the case of *h* = 0.

Static magnetic structures are obtained by simulating annealing process using Monte Carlo method. Detail discussion on the simulation is described in a method section. To obtain dynamical features, we run a simulation based on Landau-Lifshitz-Gilbert equation where the effect of the STT is added;

2 $$\frac{\partial {{\bf{S}}}_{i}}{\partial t}=-\gamma {{\bf{S}}}_{i}\times {{\bf{H}}}_{{\rm{eff}}}+\alpha {{\bf{S}}}_{i}\times (\frac{\partial {{\bf{S}}}_{i}}{\partial t})-{u}_{i}(\frac{\partial {{\bf{S}}}_{i}}{\partial x})+\beta {u}_{i}{{\bf{S}}}_{i}\times (\frac{\partial {{\bf{S}}}_{i}}{\partial x})$$

where *γ* is gyromagnetic ratio, *α* is Gilbert damping constant. The subscript *i* denotes the layer number, $-u(\frac{\partial {\bf{S}}}{\partial x})$ is an adiabatic STT, and $\beta u{\bf{S}}\times (\frac{\partial {\bf{S}}}{\partial x})$ is a non-adiabatic STT^27–29^.

## Results and Discussion

**Figure 1:**
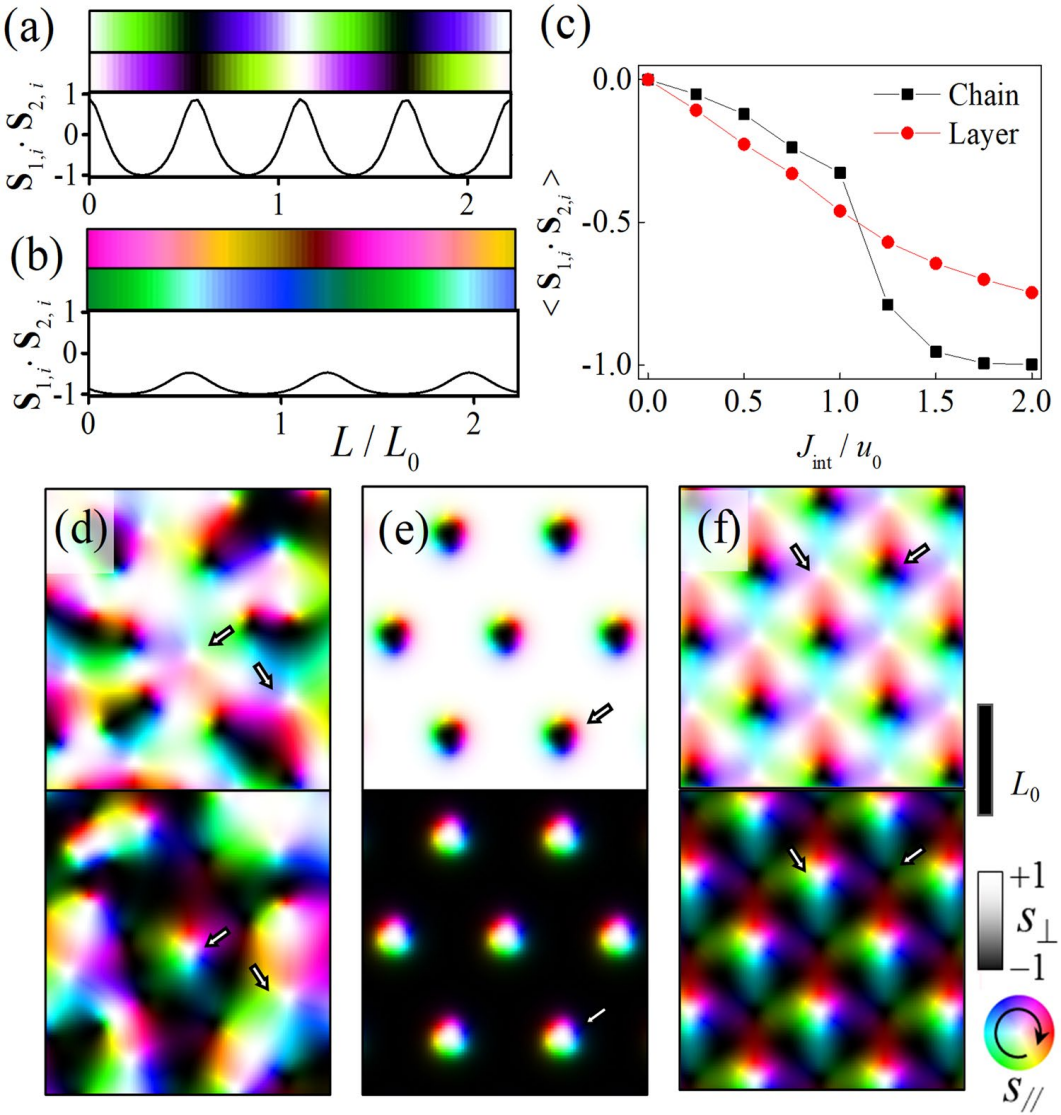
Magnetic structure of linear chains and **S**_1,*i*_∙**S**_2,*i*_ in the case of (**a**) |*J*_int_| = *u*_0_ and (**b**) |*J*_int_| = 1.25 *u*_0_. Top panel shows the spin configuration of layer 1, middle panel shows the spin configuration of layer 2, and the graph of bottom panel shows **S**_1,*i*_∙**S**_2,*i*_. (**c**) Spin correlation of the system vs. interlayer coupling strength. Red and black lines denote the spin correlation of layer and chain system. (**d**–**f**) Magnetic structures obtained under the same strength of interlayer coupling (|*J*_int_| = 2 *u*_0_). The differences are from the different initial setups of skyrmion seeds. The top panel shows the spin configuration of layer 1, bottom panel shows the spin configuration of layer 2. Arrow are used to indicate the same positions in two layers. The scale bar is *L*_0_. The color wheel and gray scale show the spin direction.

We first simulated two linear chain (or ladder) systems as a reduced dimensional case [Fig. 1(a)]. Without interlayer coupling, each spin chain makes a helical structure with a periodic length of 2π*J*/*D*. When the interlayer coupling is weak (|*J*_int_| < *u*_0_), the structures of two chains are only partially correlated. The first two rows in Fig. 1(a) shows the helical structures in chain 1 and chain 2 each, and the bottom graph shows the local correlation **S**_1_∙**S**_2_ of the spin structure. Around |*J*_int_| ~ *u*_0_, the average correlation changes abruptly indicating a phase transition. When *J*_int_ is just above the critical value, it still shows periodic structure, but forms the conical structure of which the net magnetization is opposite in two chains, as shown in Fig. 1(b). The correlation **S**_1_∙**S**_2_ at |*J*_int_| ~ *u*_0_ shows that small deviations from $-1$(anti-parallel) but does not vary in the full range from ${-}1$ to $1$, as shown in Fig. 1(b). The square data points in Fig. 1(c) shows how the average correlation $\langle {{\bf{S}}}_{1}\cdot {{\bf{S}}}_{2}\rangle$ changes with *J*_int_. The correlation changes abruptly around |*J*_int_| ~ *u*_0_ indicating a phase transition from a helical structure to a conical structure.

The spin structure in the coupled layer system is found to be different from that of coupled spin chain system. In the coupled layer system at zero magnetic field, the spin structures obtained from Monte Carlo simulation did not show a long-range ordered structure when the system was cooled down from high temperature; skyrmions are intermixed in a complex way as in Fig. (d). This is due to the fact that interlayer coupling acts as an effective field which is required to form skyrmions in a layer system and the spins can vary spatially in any direction in the ${xy}$ plane. It indicates that multiple magnetic structures can be energetically stable. The structures shown in Fig. 1(e,f) are lattices of skyrmions, examples of the stable spin structures that appear in the coupled layer systems. These structures are obtained when we applied initial skyrmion seeds in regular arrays and released using Eq. (2). In the structure of Fig. 1(e), skyrmion pairs make a hexagonal lattice, and in the structure of Fig. 1(f), skyrmions with opposite skyrmion numbers make a square lattice. The figures show only examples of possible complex magnetic structures in which helical structures and skyrmions can coexist. In the coupled layers, the averaged correlation does not change critically at |*J*_int_| ~ *u*_0_ unlike in the coupled chains [Fig. 1(c)]. However, it is not because the layered spin structures show a transition-less variation with *J*_int_, but because many complex spin structures can be easily intermixed and coexist while the averaged correlation varies continuously.

As shown in Fig. 1(e,f), a skyrmion can exist as a stable state even without an external field, due to the interlayer coupling and its effective field^30^. The effective field induced by *J*_int_ plays a role as a perpendicular field which stabilizes a skyrmion in the partner layer. The formation of skyrmions without any external magnetic field or anisotropy can be an advantage of this system in the spintronics applications.

**Figure 2:**
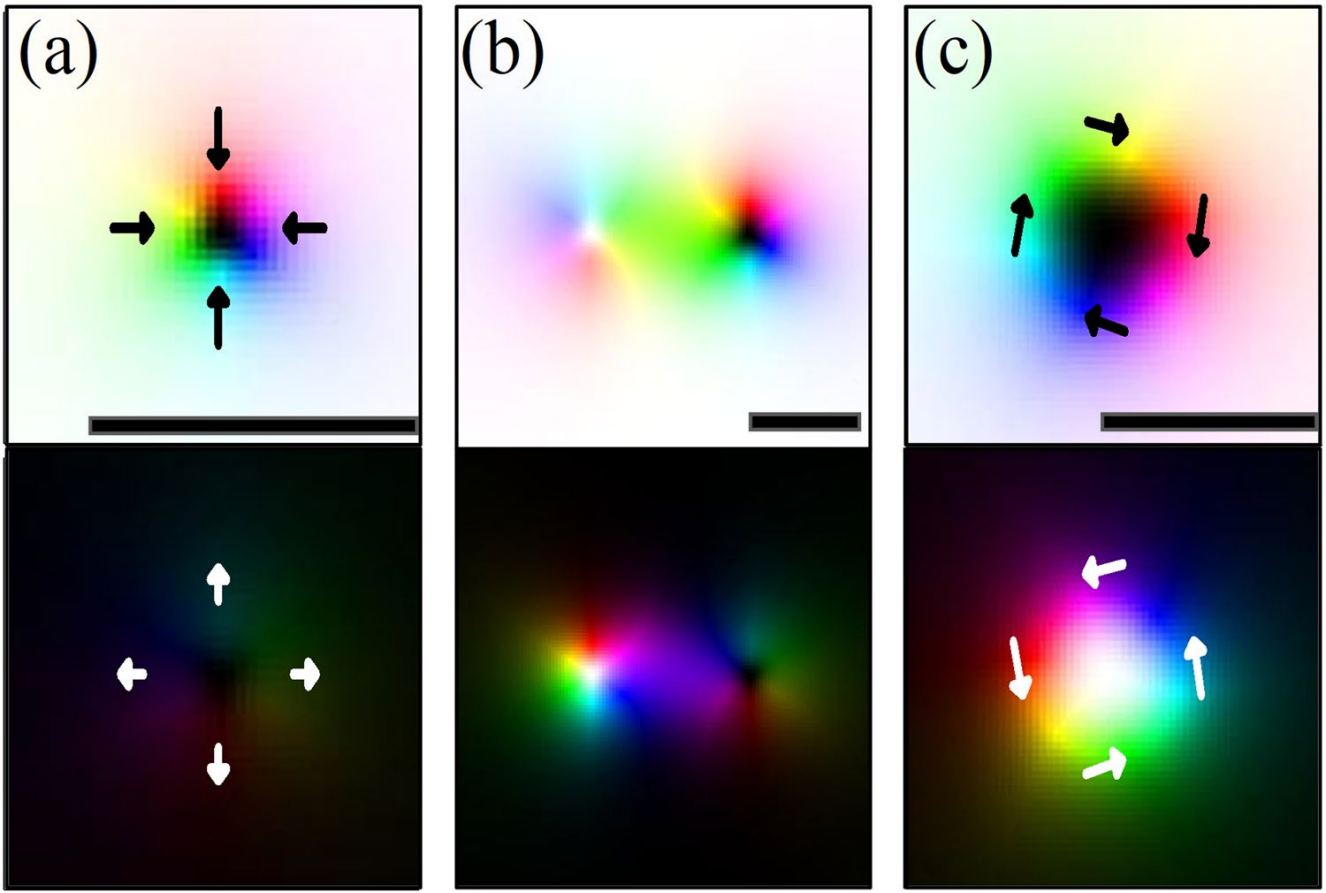
Stable skyrmion states built without an external field. (**a**) ‘Sole skyrmion’ state: a skyrmion is in only one layer. (**b**) Coupled ‘Sole skyrmion’ states: Two skyrmions are coupled to each other through in-plane magnetization bridge. (**c**) ‘Skyrmion pair’ state: It is neither a Bloch nor a Néel type skyrmion. The arrows of (**a**,**c**) indicate the magnetization direction of the rim part of the skyrmions.

We investigated the possible stable states including skyrmion. They can be classified into two cases by whether the core of a skyrmion in one layer coincides with that of other layer. Figure 2(a) shows the case where a skyrmion is in a stable state in layer 1, while layer 2 does not hold a paired skyrmion. The structure is obtained by relaxing energy from a seed structure with an initial skyrmion number, one in a layer and zero in the other layer. We refer this structure as a ‘sole skyrmion’. The spin directions in layer 2 are mostly uniform except the boundary region of the skyrmion in layer 1, therefore, the spins **S**_2_ in layer 2 are almost S_2_, *z* ~ 1. Therefore, the effective field of interlayer coupling term can be written by ${{\bf{h}}}_{{\rm{int}}} \sim |{J}_{{\rm{int}}}|\hat{z}$ from Eq. (1), and this supplies a perpendicular effective field to stabilize a skyrmion in layer 1. The spin directions of layer 1 and layer 2 in the small core region of skyrmions are parallel coupled, which is against the interlayer interaction, but overall the spins follows the favored chirality in each layer. We investigated the condition to make this sole skyrmion state stable; the interlayer interaction should be in the range from stable skyrmion is from *u*_0_ to 4*u*_0_. If |*J*_int_| < *u*_0_, an initial skyrmion becomes a helical structure, so the spatially confined round shape structure, skyrmion, is not stable. If |*J*_int_| > 4*u*_0_, a skyrmion disappears due to the strong effective field.

When a skyrmion of one layer is coupled to that of the other layer, there can be two possible states. One is that the cores of two skyrmions are spatially separated, as shown in Fig. 2(b). In this case, two sole skyrmion states are weakly coupled via in-plane magnetization between two sole skyrmion states. In the state of Fig. 2(b), the core positions of two interacting skyrmions are separated, and the two skyrmions are located at a certain distance which reduces the interaction energy most. This interaction is not necessarily confined only in the two skyrmions, but generally allowed with neighboring skyrmions; therefore, interacting skyrmions make the lattice as shown in Fig. 1(f). Another is the directly coupled pair of two skyrmions which have cores at the same position as in Fig. 2(c). This direct type of skyrmion pairs builds the lattice structure as shown in Fig. 1(e). We refer it as a ‘skyrmion pair’. In this case, core area is wider than a sole skyrmion and has a thin in-plane part which is partially chiral. The in-plane part has tilted magnetism carrying both Bloch-type magnetization due to the interlayer interaction and the chiral Néel-type magnetization due to the DM interaction. Therefore, this structure is neither a Néel nor a Bloch type skyrmion. Though a sole skyrmion becomes unstable if |*J*_int_| > 4*u*_0_, because the effective fields from other layer becomes too strong, the skyrmion pair is still stable in the range of |*J*_int_| > 4*u*_0_, since their cores are also coupled by interlayer interaction and the effective fields supports the core magnetization. Therefore, in the range of |*J*_int_| > 4*u*_0_, only the skyrmion pair state is energetically stable and the sole skyrmion state is not. In the range of *u*_0_ < |*J*_int_| < 4*u*_0_, the structures in Fig. 2(b,c) are both energetically stable, hence both exist together and are mixed resulting in complex magnetic structures such as those shown in Fig. 1(d).

The dynamics of a skyrmion pair and a sole skyrmion under a STT is very different from each other. It is known that the skyrmion pair can move directly along the current direction without SkHE, because the skyrmion number is canceled by the sum of two skyrmion numbers. Here, we find that additionally, the motion of a sole skyrmion and the motion of a skyrmion pair have distinctive characteristics, depending on whether the motion is caused by adiabatic or non-adiabatic STT. To clearly show the difference between adiabatic and non-adiabatic STTs, we only applied one of them in the simulation introducing an additional velocity term *ν*(=*uβ*/*α*) related to the non-adiabatic torque. Therefore, the portion of *u* and *v* estimates the contribution of the adiabatic and the non-adiabatic STT in skyrmion dynamic, respectively. The adiabatic STT contributing to the spin dynamics is $-u(\frac{\partial {\bf{S}}}{\partial x})$ and the non-adiabatic STT contribution is $\alpha v{\bf{S}}\times (\frac{\partial {\bf{S}}}{\partial x})$.

**Figure 3:**
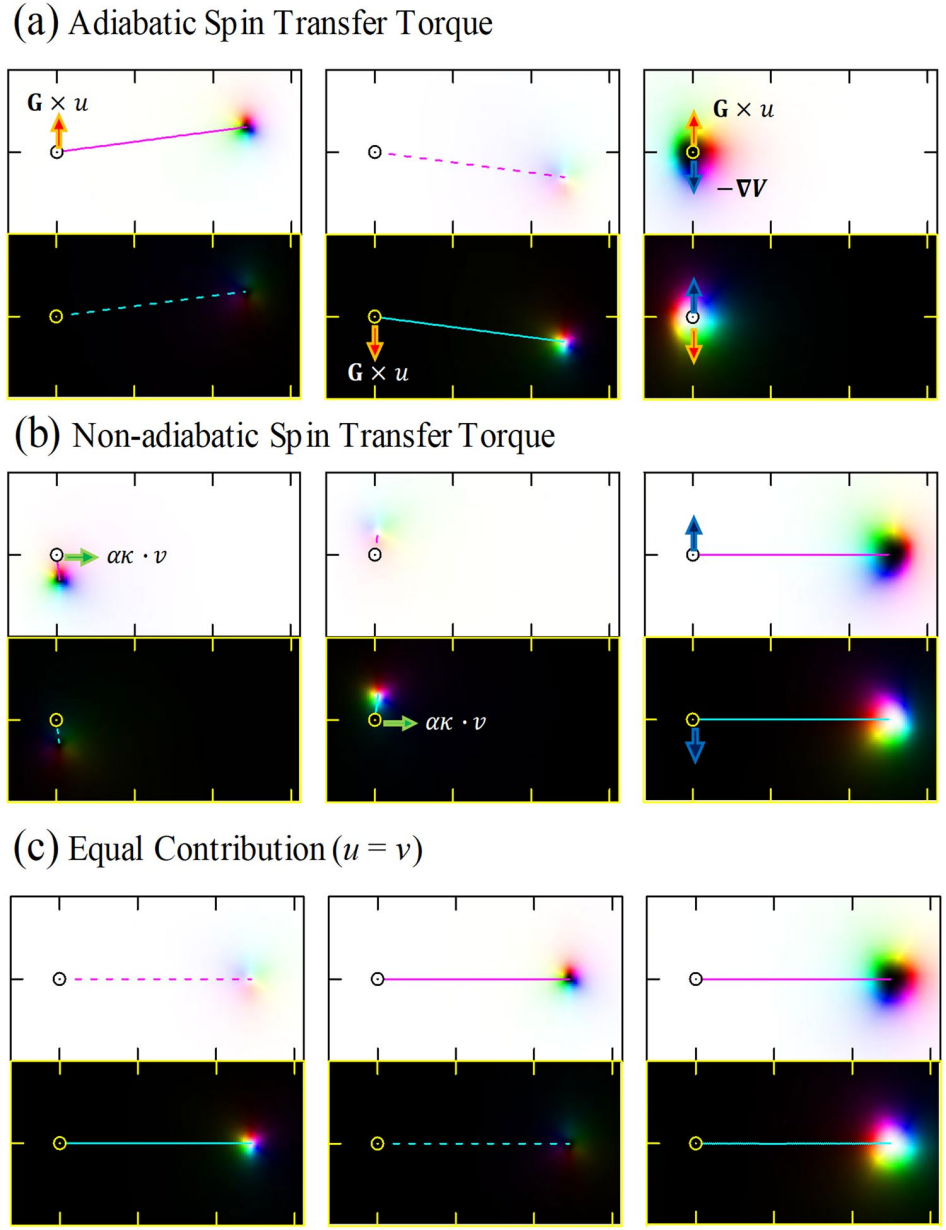
Skyrmion trajectories of ‘Sole skyrmion’ with *G* = 4*π* [left], ‘Sole skyrmion’ with *G* = 4*π* [middle] and ‘Skyrmion pair’[right], at (**a**) identical adiabatic STTs(*u*_1_ = *u*_2_ = *u* and *ν*_1_ = *ν*_2_ = 0), (**b**) identical non-adiabatic STTs(*u*_1_ = *u*_2_ = 0 and *ν*_1_ = *ν*_2_ = *ν*), and (**c**) equal contribution of adiabatic and non-adiabatic STTs (*v* = *u*). All STTs are along *x*-direction. Circular dots are for the original position of the magnetic structure in the steady state. Solid lines are the trajectories of skyrmions and dashed lines are those of non-skyrmion magnetic structure. Trajectory lines are obtained by calculating the center of skyrmion. Red arrows denote the force induced by adiabatic STT, green arrows denote the force induced by non-adiabatic STT, and blue arrows denote the force induced by the *J*_int_.

Figure 3(a) compares the dynamics of a sole skyrmion and skyrmion pair under identical adiabatic STTs in each layer (*u*_1_ = *u*_2_ = *u*, *ν*_1_ = *ν*_2_ = 0). Under the adiabatic STT, a sole skyrmion moves in a diagonal direction to the current direction *u* showing the SkHE. The SkHE of the sole skyrmion in layer 1 (left panel in Fig. 3(a)) and the SkHE of the sole skyrmion in layer 2 (middle panel in Fig. 3(a)) is opposite since the skyrmions have opposite chirality. In the case of two sole skyrmion state weakly coupled by in-plane edges (Fig. 2(b)), each skyrmion is found to follow the dynamic of a sole skyrmion and soon be separated, not affected much from other skyrmion. Though two skyrmions are coupled via in-plane magnetization, this coupling was not strong enough to cause different dynamic.

However, the skyrmion pair (Fig. 2(c)) showed very different dynamics; it stays still, not moving at all under the same condition, so we can see that the adiabatic STT cannot activate a motion of the skyrmion pair. The skyrmion in each layer has a skyrmion number of opposite sign. Therefore, the forces produced by the adiabatic STT [Red arrows in figures] are opposite and the directions of SkHE are opposite as well. For interlayer coupling strong enough to bound the two skyrmions, the force induced by the *J*_int_ is opposite to the SkHE direction. It attracts the partner skyrmion to prevent the increase of the interlayer interaction energy [blue arrows in Fig. 3(a)]. As the forces from *J*_int_ and adiabatic STT are opposite, they act against to each other and the net force on a skyrmion is canceled out.

The motions under the identical non-adiabatic STTs (*u*_1_ = *u*_2_ = 0, *ν*_1_ = *ν*_2_ = *ν*) in each layer are represented in Fig. 3(b). In this case, a sole skyrmion shows motion nearly perpendicular to the current direction; however, the magnitude of the displacement is much smaller compared to the adiabatic STT induced motion and the tilting direction is opposite to the direction of the adiabatic STT case. The directions of SkHE induced by the non-adiabatic STT of each layer are also opposite due to the different sign of the skyrmion number, but the forces *ακ*∙*ν* from non-adiabatic STT are in the same direction[Green arrows in Fig. 3(b)].

On the other hand, The skyrmion pair drifts along the direction of *v*, faster than the sole skyrmion. In this case, interlayer interaction also provides an attractive force to prevent the decoupling of skyrmion pair. Since the force from *J*_int_ is perpendicular to the force from the non-adiabatic STT, it is not canceled out and produces a non-zero torque. Though the directions of forces on two skyrmions are opposite to each other, the gyrodynamic motion propels each skyrmion in the same direction because of their opposite skyrmion number. It needs to be noted that the torques propelling skyrmions are not the STT but the attraction force binding two skyrmions against SkHE. Therefore, the gyrodynamic motion produces a fast drift, even though the force from STT is not strong. And the drift motion is perpendicular to the motion caused by SkHE that is perpendicular to the current direction, therefore, the skyrmion pair moves along the current direction.

In the case when the both STTs equally contribute (*u* = *ν*) [Fig. 3(c)], The motions of a sole skyrmion and a skyrmion pair become similar to each other.

We compared our simulation results with the analysis with Thiele equation^31–33^ which is a standard tool to analyses the skyrmion or vortex motion, especially, for the paired skyrmion or vortices under the action of STT effect. The Thiele equations for the skyrmions under STT are

3 $$\begin{array}{c}{{\bf{G}}}_{1}\times ({{\bf{u}}}_{1}-{\dot{{\boldsymbol{r}}}}_{1})+\alpha {\kappa }_{1}\cdot ({{\bf{v}}}_{1}-{\dot{{\boldsymbol{r}}}}_{1})-\nabla {V}_{12}=0\\ {{\bf{G}}}_{2}\times ({{\bf{u}}}_{2}-{\dot{{\boldsymbol{r}}}}_{2})+\alpha {\kappa }_{2}\cdot ({{\bf{v}}}_{2}-{\dot{{\boldsymbol{r}}}}_{2})-\nabla {V}_{21}=0\end{array}$$

where $\dot{{\bf{r}}}$ is the drift velocity of skyrmion, *α* is the Gilbert damping constant, and *V*_*ij*_ is the potential induced by the other layer. **G**_*i*_ and *κ*_*i*_ are gyromagnetic coupling vector and dissipative dyadic tensor of the magnetic structure each layer. The directions of *u*_i_ and *v*_i_ are *x*-direction. The gyromagnetic coupling vectors and the dissipative dyadic tensors are related to the topological properties of the magnetic structure and their magnitudes are ${\rm{G}}=\int {\bf{S}}\cdot ({\partial }_{x}{\bf{S}}\times {\partial }_{y}{\bf{S}})$ and $\kappa =\int {\partial }_{x}{\bf{S}}\cdot {\partial }_{x}{\bf{S}}\cong \int {\partial }_{y}{\bf{S}}\cdot {\partial }_{y}{\bf{S}}$.

For the case of a sole skyrmion, **G**_1_ = *G*, *κ*_1_ + *κ*_2_ = *κ*_tot_ and the solution is

4 $$\begin{array}{c}{\dot{x}}_{1}=\frac{{G}^{2}}{{G}^{2}+{\alpha }^{2}{{\kappa }^{2}}_{{\rm{tot}}}}{u}_{1}+\frac{{\alpha }^{2}{{\kappa }^{2}}_{{\rm{tot}}}}{{G}^{2}+{\alpha }^{2}{{\kappa }^{2}}_{{\rm{tot}}}}{v}_{1}\\ {\dot{y}}_{1}=\frac{\alpha {\kappa }_{{\rm{tot}}}G}{{G}^{2}+{\alpha }^{2}{{\kappa }^{2}}_{{\rm{tot}}}}{u}_{1}-\frac{\alpha {\kappa }_{{\rm{tot}}}G}{{G}^{2}+{\alpha }^{2}{{\kappa }^{2}}_{{\rm{tot}}}}{v}_{1}\end{array}$$

The equation can be approximated as ${\dot{x}}_{1}={u}_{1}$ and ${\dot{y}}_{1}=0$ especially when *α* is small ($\alpha  <  < \frac{{\rm{G}}}{\kappa }$). From the equations, we can see that the motion of a sole skyrmion is mostly dependent on the adiabatic STT. If a skyrmion is in layer 1 only, the STT on layer 2 has little influence on its motion. The motions along $\hat{x}$ caused by *u* and *ν* are same along the current direction, but the motion along $\hat{y}$ by SkHE is opposite for *u* and *ν*. It is important because the direction of the interaction force −∇*V* induced by the interlayer interaction is opposite to the direction of the force separating the coupled skyrmion induced by the current and the produced torques from the interaction forces are opposite as well.

In a strongly coupled skyrmion pair in a weak current, the spins in pair sites are all opposite and move together (***r***_1_ = ***r***_2_); thus, **G**_1_ = −**G**_2_ = *G*, *κ*_1_ = *κ*_2_ and the interaction between two skyrmion acts to the opposite direction ∇*V*_12_ = −∇*V*_21_. Therefore, the total gyromagnetic coupling vector **G**_tot_ is zero and total dyadic tensor *κ*_tot_ is 2*κ*_1_ (or 2*κ*_2_). When two skyrmions are strongly coupled and move together, the drift velocity of a strongly coupled skyrmion pair is

5 $$\begin{array}{c}\dot{x}=\frac{{v}_{1}+{v}_{2}}{2},\\ \dot{y}=\frac{G}{\alpha {\kappa }_{{\rm{tot}}}}({u}_{1}-{u}_{2}).\end{array}$$

The interaction force acting on a skyrmion of layer 1 in the skyrmion pair is

6 $$\begin{array}{c}{F}_{x,1}=-\frac{\partial V}{\partial x}=-\frac{{G}^{{\rm{2}}}}{\alpha {\kappa }_{1}}(\frac{{u}_{1}-{u}_{2}}{2})-\alpha {\kappa }_{1}(\frac{{v}_{1}-{v}_{2}}{2}),\\ {F}_{y,1}=-\frac{\partial V}{\partial y}=G(\frac{{v}_{1}+{v}_{2}}{2}-\frac{{u}_{1}+{u}_{2}}{2}).\end{array}$$

If the STT in layer 1 and 2 are identical(*u*_1_ = *u*_2_ = *u* and *ν*_1_ = *ν*_2_ = *ν*), The force along the current direction is zero (*F*_*x*,1_ = −*F*_*x*,2_ = 0) and the force along the current direction is *F*_*y*,1_ = −*F*_*y*,2_ = G(*ν*−*u*). Therefore, the forces from the adiabatic STT and the non-adiabatic STT have opposite direction to each other. When the skyrmions of each layer are under STTs, the SkHE along y-direction makes both skyrmions separate from each other. The interlayer interaction, however, is strong enough to maintain the skyrmion pair, hence the origin of the force in Eq. (6) is the restoring force to overcome opposite gyrodynamic motion caused by the opposite skyrmion number.

Since the equation of motion is ${\rm{G}}{\dot{x}}_{1}={\rm{G}}{u}_{1}-\frac{\partial V}{\partial {y}_{1}}-\alpha {\kappa }_{1}{\dot{y}}_{1}={\rm{G}}u+G(v-u)-0={\rm{G}}v$, the interaction force caused by the adiabatic STT, −*Gu* is canceled out with the direct force from adiabatic STT, *Gu*. Therefore, the net force on skyrmion pair caused by the adiabatic STT is zero and the motion along the current direction is only affected by the interaction force −*Gν* which is originated from non-adiabatic STT. As a result, the propelling torques for a skyrmion pair and a sole skyrmion is distinctive. The former is non-adiabatic STT, and the latter is adiabatic STT.

If the STT in two layers are not identical, the motion can be described by Eq. 6 in general. Especially when the STTs are exactly opposite to each other (*u*_1_ = −*u*_2_ and *ν*_1_ = −*ν*_2_), only *F*_*x*_ contributes and skyrmion pair moves in the y direction. In this case, the propelling torque for a skyrmion pair becomes the adiabatic STT.

**Figure 4:**
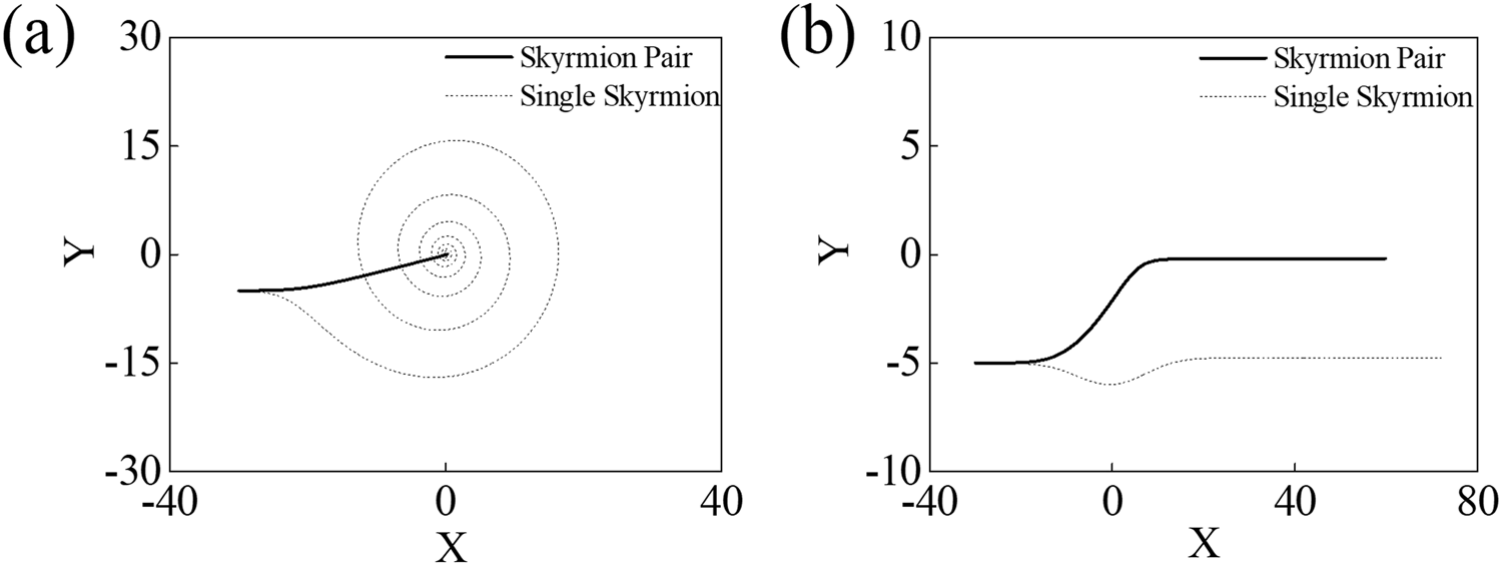
Trajectories of a single skyrmion and a skyrmion pair influenced by an attractive pinning potential *U*(*x*, *y*) = −*U*_0_exp[−(*x*^2^ + *y*^2^)/100] were obtained by numerical calculations. Trajectories under (**a**) strong pinning potential(*U*_0_ = 50) and (**b**) weak potential (*U*_0_ = 0.2) are represented with lines. Solid lines denote trajectories of a skyrmion pair and dashed lines denote those of a single skyrmion. The initial position is set at **r**_0_ = (−30, −5).

The dynamics of a skyrmion is influenced by pinning potentials, though it is robust and less affected by impurities or non-adiabatic effect than other magnetic structures^34–36^. Although a skyrmion pair moves only with non-adiabatic STT, it is not because of the direct effect from non-adiabatic STT, but because of the attractive interaction between the two skyrmions. Hence, the dynamics of the skyrmion pair is influenced by impurities as much as a single skyrmion is. Since the total gyromagnetic coupling vector, G, of a skyrmion pair is zero, the detailed dynamics from a pinning potential is different: a skyrmion pair moves straight while a sole skyrmion moves spirally around the attraction center (Fig. 4).

**Figure 5:**
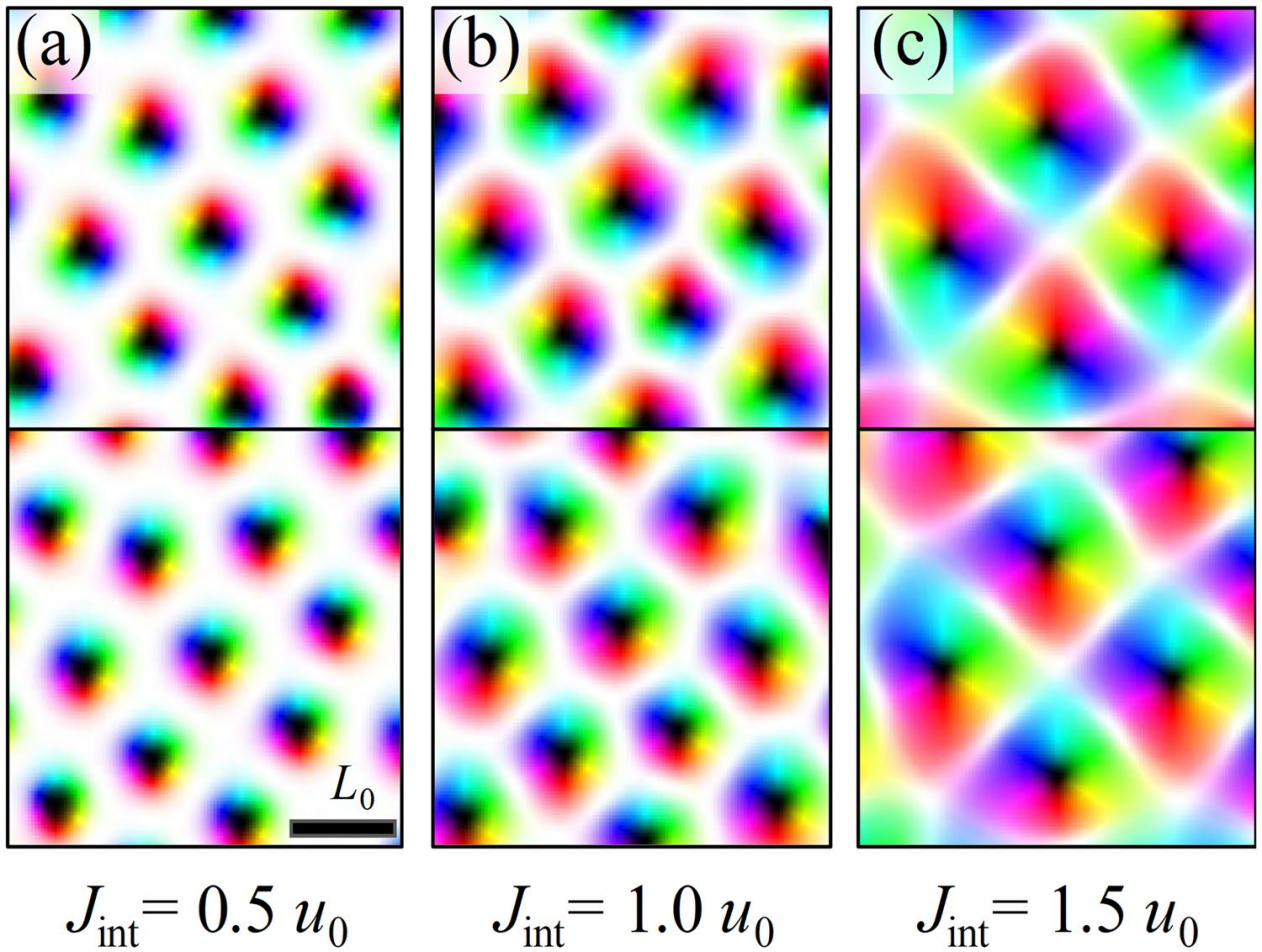
(**a**–**c**) Magnetic structures for the case of *h* = *u*_0_ and |*J*_int_| = 0.5*u*_0_, *u*_0_, 1.5*u*_0_. The top figures of (**a**–**c**) represent the structures of the layer 1, and the bottom figure represents those of the layer 2. Skyrmion lattices appear in the figures.

When an external field *h* ~ *u*_0_ is applied, the paired (coupled) skyrmion lattice structure can be stabilized (Fig. 5). Note that the skyrmion pair is not the same kind of skyrmion pair in Fig. 2. Under external field(*h* ~ |*J*_int_|), skyrmions composing a pair with the same skyrmion number, because the magnetic field overcome the interlayer interaction and the skyrmions have same magnetic background (shown as white color in figure). The simulation results show that the spins in the core region of the paired skyrmions are parallel but the spins in the rim of a skyrmion in layer 1 is antiferromagnetically coupled with that of the skyrmion in layer 2 to reduce the *J*_int_ energy. Therefore, the skyrmions in two layers have the same polarity but opposite chirality. As *J*_int_ is increased, the area of the rim (or boundary) region of the coupled skyrmion, which have opposite in-plane magnetizations in the top and bottom layers, increases to reduce the interlayer coupling energy. Therefore, the size of skyrmions increases with *J*_int_ accordingly (Fig. 5). When the interlayer coupling is weak (|*J*_int_| < *u*_0_) or moderate (|*J*_int_| ~ *u*_0_), skyrmions form a coupled hexagonal lattice with matching skyrmion lattice sites in the top and bottom layers [Fig. 5(a,b)]. The skyrmions in the top and bottom layers have opposite chirality with the in-plane boundary regions having opposite magnetization and the core regions having the same polarity. When *J*_int_ is strong (|*J*_int_|>>*u*_0_), skyrmion shape becomes irregular and the long-range order forming lattice structure is reduced [Fig. 2(c)]. The strong *J*_int_ increases area of the rim (or boundary) of skyrmions where the spin profile is flattened, thus the role of DM interaction causing regular chiral structure is reduced.

**Figure 6:**
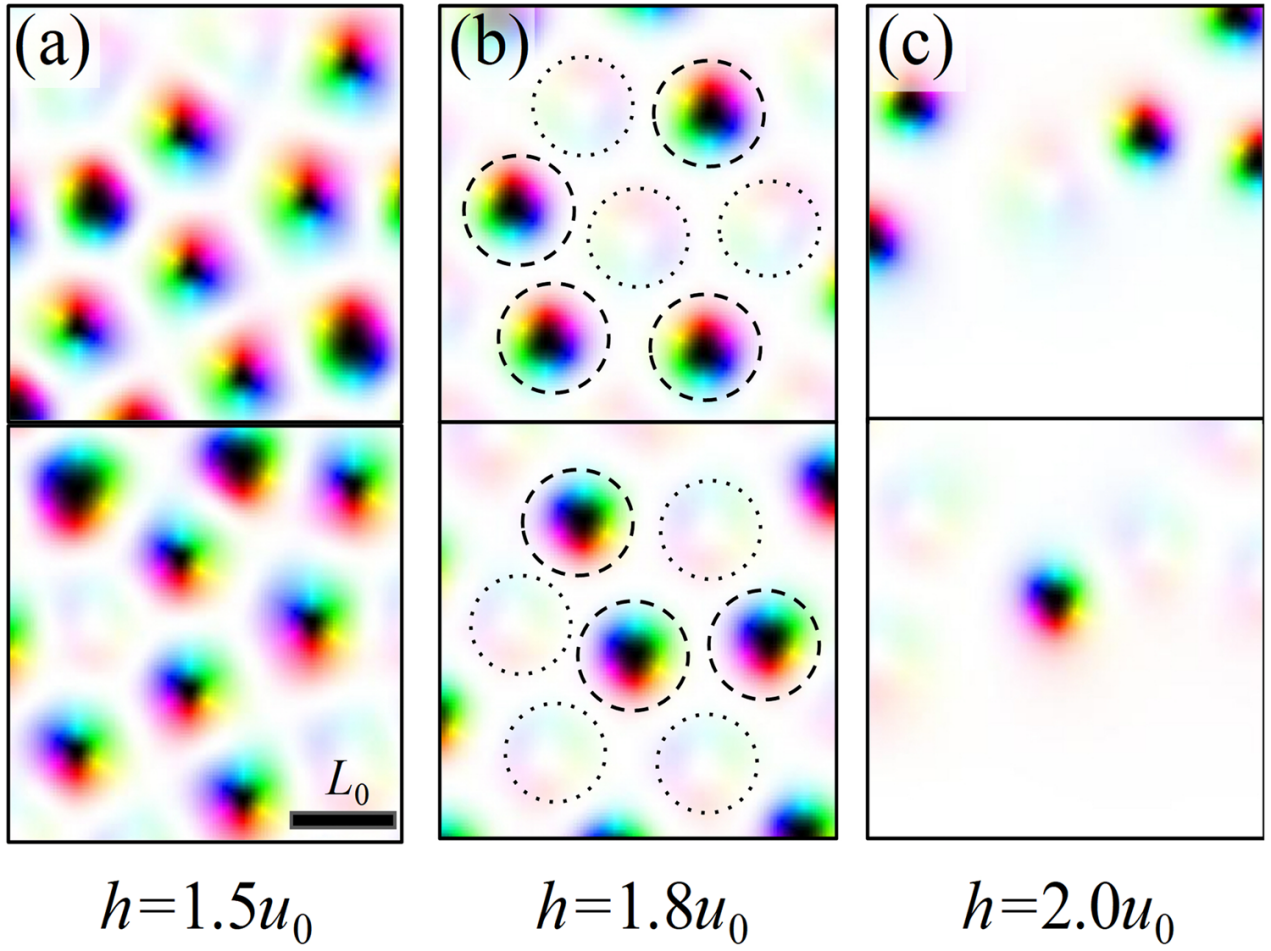
(**a**–**c**) Magnetic structures for the case of |*J*_int_| = 0.5*u*_0_ and *h* = 1.5*u*_0_, 1.8*u*_0,_ 2*u*_0_. In figure (**b**), dashed line denotes residual skyrmions of coupled skyrmion at each lattice point and short-dashed line denotes extinguished skyrmions of that. Lattice points represented by dashed and short dashed line form hexagonal lattice through layer 1 and 2.

As the external field is increased, some of the skyrmions are annihilated. The series of figures [Fig. 6(a–c)] shows how the skyrmions are extinguished. Since two layers are identical (*J*_1_ = *J*_2_ and |**D**_1_| = |**D**_2_|), the annihilation process is random: skyrmions in top layers are extinguished at some lattice sites while skyrmions in the bottom layer are extinguished in other lattice site. In Fig. 6(a), the behavior is somewhat exclusive at this stage, so a skyrmions in one layer is extinguished while the skyrmion in other layer is preserved. As one skyrmion of the coupled skyrmion is annihilated, another skyrmion gets to occupy more area and the regularity of the hexagonal order is broken [Fig. 6(a)]. The extinguishment of the skyrmion in one layer reduces the frustration caused by antiferromagnetic interlayer interaction and stabilizes the now unpaired skyrmion in the other layer. This results in the remaining skyrmion being stabilized and becoming larger. At a certain external field (Fig. 6(b)), every skyrmion lattice site has only one skyrmion either in the layer 1 or the layer 2. A regular hexagonal structure is rebuilt if both layers are combined, though the skyrmions in each layer do not make a regular structure. Since one skyrmion lattice site can be considered as one bit of memory/information, this exclusive nature can be potentially useful for spin logic operation such as ‘inverse’ or ‘exclusive or’, especially when considering the fact that the information of two near lattice sites within a layer does not interfere with each other. When the magnetic field if further increased, the remaining skyrmion become extinguished as well and the hexagonal order through both layers is broken again [Fig. 6(c)].

## Conclusion

We investigated the skyrmion structures and their dynamics in interlayer coupled systems when the chirality are opposite in two layers. Skyrmions and skyrmion pairs are stable state without an external field. A skyrmion pair exhibits different dynamics from a sole skyrmion without pairing another skyrmion in the other layer. When the STT in two layers are identical, the former is propelled by skyrmion-skyrmion interaction and moves along the spin current, while the latter is mostly propelled by adiabatic STT and moves in the oblique direction. Under an external field, skyrmions in the skyrmion lattice disappear in an exclusive way in which a skyrmion in one layer is extinguished prior to that of another layer during the magnetic saturation process.

## Methods

In simulation, we used square spin grids composed of ~10^6^ spins interacting with neighborhood four spins via exchange interaction and DMI (Eq. 1). We did not consider magnetic anisotropy or magnetic dipole interaction, and the parameters of each layer were set to be identical except the sign of the DM interaction in order to simplify the scopes of our study. The antiferromagnetic interlayer coupling case is studied; however, generalization to the ferromagnetic case can be done by substituting **S**_2_→−**S**_2_ in the case of *h* = 0. The direction of a DM vector is set to perpendicular to **r**_ij_^37,38^, but can also be generalized to the parallel case(**D**_ij_||**r**_ij_)^39,40^ by *S*_*x*_→*S*_*y*_, *S*_*y*_→−*S*_*x*_, where **r**_ij_ is the displacement vector between *i* and *j* spins. Therefore, some of our results and discussion are valid for ferromagnetic interlayer coupling case and other types of DM interaction. However, it should be noted that the case of an opposite chirality cannot be converted to the case of an identical chirality by such a simple symmetry operation.

When only a layer with *J* and *D* is considered, the periodic length of a chiral structure is proportional to *J*/*D* and the effective field from DM interaction to form chiral structure such as a skyrmion lattice is proportional to *D*^2^/*J*^19^. Therefore, we use *J*/*D* to discuss the periodic length of the chiral structure and *D*^2^/*J* to discuss the strengths of effective fields. In our simulation, we typically used *J* = 1 and *D* = 0.2, however, the values do not affect results after we convert length and field units to *L*_0_ = 2*πJ*/*D* and *u*_0_ = *D*^2^/*J* as long as *D*<<*J*. In the continuum approximation, the energy density unit becomes ${u}_{0}=\frac{{{D}_{M}}^{2}}{2A}$ and length unit becomes ${L}_{0}=4\pi \frac{A}{|{D}_{M}|}$, where *A* is the exchange stiffness and *D*_*M*_ is the DMI strength. The real parameters for typical DM system are listed by *L*_*D*_ = *L*_0_, related to the periodic length of chiral structure, and *h*_*D*_ = **M***u*_0_, related to the required field strength to form skyrmion lattice. Here, *a* is a lattice constant, and **M** is the magnetization vector^41^. As an example, *L*_*D*_ and *h*_*D*_ of FeGe are 70 nm and 0.4 T.

To investigate the magnetic ground state or stable state, we run Monte Carlo simulations based on a heat bath method^42^. In the method, a temperature parameter was slowly decreased maintaining the thermally stable state at specific external field strength, until we obtain a stable state which does not vary with further iterations any more. In the stable states, every spin is along its local effective field ${{\bf{H}}}_{i,\mathrm{eff}}=-{\partial U/\partial {\bf{S}}}_{{\rm{i}}}$. To make structures with definite skyrmion numbers (Fig. 1(e,f) and Fig. 2), we started the simulation from initial structures having skyrmion numbers same as the final stable structures. To investigate the dynamic feature of skyrmion such as the motion under spin transfer torques, we numerically solved LLG equation (Eq. 2) and the motions obtained from the simulation are compared with the analytic solution from the Thiele equation(Eq. 3).
